# Involvement of DNA-PKcs in the Type I IFN Response to CpG-ODNs in Conventional Dendritic Cells in TLR9-Dependent or -Independent Manners

**DOI:** 10.1371/journal.pone.0121371

**Published:** 2015-03-26

**Authors:** Chi Ma, Narrissa P. Spies, Ting Gong, Can Xin Jones, Wen-Ming Chu

**Affiliations:** Department of Cancer Biology, University of Hawaii Cancer Center, Honolulu, Hawaii 96813, United States of America; Istituto Superiore di Sanità, ITALY

## Abstract

CpG-ODNs activate dendritic cells (DCs) to produce interferon alpha (IFNα) and beta (IFNβ). Previous studies demonstrated that Toll-like receptor 9 (TLR9) deficient DCs exhibited a residual IFNα response to CpG-A, indicating that yet-unidentified molecules are also involved in induction of IFNα by CpG-A. Here, we report that the catalytic subunit of DNA-dependent protein kinase (DNA-PKcs) but not Ku70 deficient BMDCs showed defective IFNα and IFNβ responses to CpG-A or CpG-B. Loss of both DNA-PKcs and TLR9 further reduced the IFNα response to CpG-A. These DNA-PKcs and TLR9 effects were mediated by their downstream Akt/mTORC1 pathway and downstream events IRAK1 and IKKα. Loss of DNA-PKcs, TLR9, MyD88 or IRAK4 impaired phosphorylation of Akt(S473), S6K, S6, IRAK1, or IKKα in BMDCs in response to CpG-ODNs. The residual IFNα and IFNβ in DNA-PKcs-deficient BMDCs were partially responsible for the induction of IL-6 and IL-12 by CpG-ODNs and their stimulatory effect was blocked by IFNAR1 neutralizing antibodies. Further analysis indicated that CpG-ODN associated with DNA-PKcs and Ku70, and induced DNA-PKcs’s interaction with TRAF3. Intriguingly, DNA-PKcs but not Ku70 expression level was reduced in TLR9-deficient BMDCs. Taken together, our data suggest that DNA-PKcs is an important mediator in the type I IFN response to CpG-ODNs in TLR9-dependent or -independent fashions.

## Introduction

Bacterial and viral genomic DNA containing the CpG motif (CpG-DNA) and its analog oligodeoxynucleotides containing CpG motif (CpG-ODNs) are powerful activators of the innate immune system. There are two major types of CpG-ODNs, CpG-A and CpG-B. CpG-A prefers activating plasmacytoid DCs (pDCs), whereas CpG-B efficiently activates B cells, conventional dendritic cells (cDCs) and macrophages[1]. CpG-B strongly activates DCs and macrophages to produce pro-inflammatory IL-6 and IL-12, which is critical for the Th1 response and subsequent anti-infectious and anti-tumor activities. Also, CpG-A together with DOTAP (a lipid) triggers DCs to produce the type I IFN (IFNα and IFNβ), and CpG-B is able to induce IFNβ expression. It is known that CpG-ODNs activate TLR9, which in turn recruits the adaptor protein myeloid differentiation factor 88 (MyD88) and IL-1 receptor associated protein kinase 4 (IRAK4) leading to activation of IRAK1 and IKKα, which then activate the IFN regulatory factor 7, resulting in expression of the type I IFN[2, 3]. However, loss of TLR9 does not abolish the IFNα response to CpG-A, but abolishes the IFNβ response to CpG-B suggesting that an unidentified factor also mediates the IFNα response to CpG-A.

TNF receptor-associated factor 3 (TRAF3) has been found to be important for expression of type I IFNs but not pro-inflammatory cytokines in response to CpG-ODNs[3]. CpG-ODNs induce the association of TRAF3 with MyD88[3, 4]. Yet, how TRAF3 mediates the type I IFN response to CpG-ODNs is still not well understood.

The Akt/mammalian target of rapamycin complex 1 (mTORC1) pathway is also suggested to be required for the type I IFN response to CpG-A in pDCs[5]. mTORC1 is an important downstream event of Akt, and can phosphorylate the ribosome 6 (S6) kinase (S6K), which in turn phosphorylates S6[6]. The mTORC1 inhibitor rapamycin was able to inhibit both the type I IFN and pro-inflammatory cytokine responses to CpG-ODNs[5]. Knockdown of S6K diminished CpG-ODN-induced association of TLR9 with MyD88[5], indicating that Akt and S6K might act upstream of TLR9 in CpG-ODN signaling. Interestingly, chloroquine, which abolishes the activation of TLR9-dependent pro-inflammatory signaling, had no apparent inhibitory effect on Akt activation by CpG-ODN in THP1 macrophages[7]. Our results and others suggested that TLR9 was involved in Akt(S473) phosphorylation in bone marrow-derived macrophages (BMDMs) in response to CpG-ODNs [8, 9].

In addition to above relay molecules, other proteins are suggested to be transducers in CpG-ODN signaling. One of them is DNA-PKcs, which is in both the cytoplasm and nucleus of mouse cells[8]. DNA-PKcs is an essential component of double-stranded DNA break repair complex and is vital for B and T cell development[10]. A high level of anti-DNA-PKcs autoantibody is frequently detected in serum of patients with polymyositis, scleroderma, systemic lupus erythematosus (SLE), and mixed connective tissue disease[11]. It is suggested that the type I IFN plays a principal role in the development of SLE[12] indicating that DNA-PKcs might be involved in the type I IFN expression. Indeed, in a bioassay it was found that loss of DNA-PKcs impaired CpG-B-induced type I IFN response [13]. Because the bioassay is unable to tell whether IFNα or IFNβ triggers the anti-viral activity, it was unclear whether DNA-PKcs was involved in the production of IFNα or IFNβ in the study[13]. Another study suggested that DNA-PKcs is a DNA sensor for interferon regulatory factor 3-dependent innate immunity[14].

We previously reported that DNA-PKcs regulates the IL-6 and IL-12 responses to CpG-B in BMDCs in a time- and dose- dependent manner[15]. In this study, we investigated whether and how DNA-PKcs is involved in the IFNα and IFNβ responses to CpG-ODNs. Also, we determined whether Ku70, a regulatory subunit of DNA-PK, regulates the type I IFN response to CpG-ODNs, and whether inhibition of the type I IFN pathway further impairs IL-6 and IL-12 response to CpG-ODN in DNA-PKcs-deficient BMDCs. Additionally, we examined the impact of individual deficiency of DNA-PKcs or TLR9, or double deficiency of DNA-PKcs and TLR9 on the activation of the Akt/mTORC1 pathway, IKKα and IRAK1, and the type I IFN response to CpG-ODNs. We determined whether IRAK4 and MyD88 are involved in the activation of Akt and mTORC1 by CpG-ODNs. Finally, we determined the relationship between DNA-PKcs and TRAF3 in CpG-ODN signaling.

## Materials and Methods

### Mice, regents and antibodies

*Wild type* (*WT*), *DNA-PKcs* ^*-/-*^[6, 8, 15], *Ku70* ^*-/-*^, *TLR9* ^*-/-*^, *IRAK4* ^*-/-*^ *and MyD88* ^*-/-*^[6, 8, 15–17] mice were on the C57/B6/129 genetic background, and *DNA-PKcs* ^*-/-*^ */Ku70* ^*-/-*^ and *DNA-PKcs* ^*-/-*^ */TLR9* ^*-/-*^[15] mice were generated by crossing *DNA-PKcs* ^*-/-*^ with *Ku70* ^*-/-*^ or *TLR9* ^*-/-*^ mice. Animals were bred at University of Hawaii, Honolulu, Hawaii, USA. All animal work was performed based on the protocols approved by the IACUC at University of Hawaii.

CpG-ODN 1018 (CpG-B) (5′-TGACTGTGAACGTTCGAGATGA-3′) and CpG-ODN D19 (CpG-A) (5′-GpsGpsTGCATCGATGCAGGpsGpsGpsGpsG-3′)[6] were synthesized by Trilinker Biotechnology (CA, USA). CpG-ODN 1018-biotin (CpG-biotin) was purchased from Trilinker Biotechnology. DOTAP Liposomal Transfection Reagent was from Roche (Mannheim, Germany). Rapamycin was from LC Laboratories (MA, USA). Protein A/G Sepharoses were purchased from Amersham Biosciences (Uppsala, Sweden) and streptavidin-agarose beads were from Life Technologies (NY, USA).

Anti-phospho (p) antibodies (Abs) against Akt(S473), S6K(T389), S6(S235/236), IKKα/β(S176/180), IRAK1(T209), ERK1/2(T202/Y204), and Stat1(Y701) were from Cell Signaling (MA, USA). Anti-DNA-PKcs mouse monoclonal antibody (mAb) was from Neomarker (CA, USA); anti-Ku70 mAb, anti-ERK1/2 polyclonal (pAb), and anti-TRAF3 pAb were from Santa Cruz Biotech (CA, USA); anti-IFNAR1 and isotype Abs were from Biolegend (CA, USA); and Alexa fluorescence-conjugated secondary Abs were from Life Technologies (NY, USA).

### Bone marrow-derived dendritic cell culture

As described previously[15], bone marrow cells were isolated from femurs and tibias of mice, seeded at 2–3×10^5^ cells/ml, and then cultured with 10% fetal bovine serum (FBS, Life Technologies, NY, USA) RPMI1640 medium supplemented with granulocyte/macrophage-colony stimulatory factor [GM-CSF, Biolegend (CA, USA), 20 ng/ml] for 7 days. On day 2 and day 4, 5 ml of fresh 10% FBS RPMI1640 medium containing GM-CSF (20 ng/ml) were added to the cultures. On day 7, BMDCs in culture medium were harvested, and cells remained in dishes were collected by gently rinsing with 1× phosphate buffered saline (PBS) for three times. BMDCs in both medium and rinsed PBS were combined and used for the following experiments.

### ELISA assay

BMDCs harvested on day 7 were seeded in 96-well plates at 1 or 2x10^5^/well in triplicate with 10% FBS RPMI1640 medium. DOTAP was pre-incubated with CpG-A for 30 minutes at room temperature at 10 μl per μg of DNA. The cells were treated with CpG-ODNs or left untreated for 12 or 24 hours. The levels of IL-6, IL-12p40 or IFNβ in the supernatants were determined by enzyme-linked immunosorbent assay (ELISA) via respective cytokine ELISA kits (Biolegend, CA, USA; PBL Assay Science, NJ, USA) according to the manufacturer’s manual. The level of IFNα was detected by ELISA using kits from PBL Assay Science (NJ, USA) according to manufacturer’s instruction.

### Immunostainning for flow cytometry

After 7-day culture, 0.5–1×10^6^ BMDCs were stained with PE-conjugated CD11c, FITC-conjugated MHC-II-I-A/I-E and APC-conjugated CD11b (Biolegend, CA, USA) in PBS plus 1% BSA (staining buffer) for 1 hour at 4°C, and washed 3 times with staining buffer, then fixed with 1% paraformaldehyde in PBS. The samples were analyzed on a FACSCalibur (BD, CA, USA) and processed with Flowjo software.

For intracellular staining, 1×10^7^ BMDCs were seeded in petri dishes and treated with CpG-B (1μM), CpG-A (3μM) with DOTAP, or left untreated for 24 hours. After the cells were stained with PE-conjugated CD11c, APC-conjugated MHC-II-I-A/I-E and PE-Cy7-conjugated CD11b (Biolegend, CA, USA), cells were fixed with 1% paraformaldehyde in PBS in the dark for 20 minutes at room temperature. Fixed cells were resuspended in Permeabilization Wash Buffer (Biolegend, CA, USA), centrifuged at 350x g for 5 minutes, and then washed with Permeabilization Wash Buffer twice. BMDCs were stained with either FITC-conjugated IFN-α or FITC-conjugated IFN-β (PBL Assay Science, NJ, USA) for 30 minutes at room temperature, and fixed with 1% paraformaldehyde in PBS. The samples were analyzed on a BD LSRFortessa (BD, CA, USA) and analyzed with Flowjo software. Only CD11c^+^CD11b^+^MHC-II^+^ cells were analyzed for the IFNs expression.

### Immunoblotting and immunoprecipitation assays

BMDCs were harvested, washed with 1x PBS twice and starved for 2 hours in pre-warmed serum-free RPMI-1640 medium (SFM). The cells were treated with CpG-B (1μM) or CpG-A (3μM) in pre-warmed SFM for the indicated durations or left untreated. Whole cell lysates (WCLs) were prepared in the lysis buffer (1% Triton-X100)[15] and 60 μg proteins were separated on 10% SDS-PAGE and transferred onto PVDF membranes. The membranes were blocked with 5% non-fat milk in PBS containing 0.1% Tween 20 or SuperBlock®T20 blocking buffer (Thermo-Pierce, IL, USA) for 1 hour and probed with correspondent primary Abs and secondary Abs-conjugated with horseradish peroxidase (HRP), and detected by enhanced chemiluminescence (ECL, Thermo-Piers, IL, USA).

For immunoprecipitation (IP) assays[6, 8, 15], after starvation, BMDCs were treated with CpG-B (1μM) in pre-warmed SFM for the indicated durations or left untreated, and then lysed with the lysis buffer (1% NP-40). WCLs (400μg) were pre-cleared with 40 μl of protein A/G Sepharose (beads) (1:1) for 30 minutes. Pre-cleared WCLs were incubated with anti-DNA-PKcs Ab (0.8μg) or control mouse IgG (0.8μg) together with 50 μl of protein A/G beads at 4°C overnight with rotation. The beads were washed four times with the lysis buffer (0.1% NP-40) containing 1 mM phenylmethylsulfonylfluoride (PMSF, Sigma, USA). Proteins with beads were boiled, loaded on 10% SDS-PAGE and transferred onto PVDF membranes followed by immunoblotting (IB) analysis.

### Pull-down assay

For pull-down assays, starved BMDCs were treated with CpG-biotin (1μM) for 10, 30 minutes or left untreated and then washed twice with cold 1× PBS[15]. Cells were lysed with the lysis buffer [1% digitonin (Sigma, USA)][18]. WCLs (400 μg) were pre-cleared with protein A/G beads, incubated with 22 μg CpG-biotin for 2 hours, and then with 50 μl streptavidin-agarose beads at 4°C overnight with rotation. The beads were washed four times with the lysis buffer (0.1% digitonin, 1 mM PMSF), boiled, separated on 10% SDS-PAGE, and transferred onto PVDF membranes followed by IB analysis.

### Immunofluorescence staining

The immunofluorescence staining was described previously[6, 8, 15]. Briefly, harvested BMDCs were seeded in 8-well chamber slides at 2×10^5^ cells/chamber and cultured with 10% FBS RPMI-1640 medium overnight. Cells were starved for 2 hours in pre-warmed SFM and then treated with CpG-B (1μM) for 30 minute or left untreated. Cells were fixed with 4% paraformaldehyde, permeabilized with 0.2% Triton X-100, blocked with 5% BSA in PBS, and stained with primary anti-DNA-PKcs and anti-TRAF3 Abs in PBS with 2% BSA at 4°C overnight. Alexa fluorescence-conjugated secondary Abs (Alexa 488 or 594) (Lifetechnologies, NY, USA) was added and incubated for 2 hours. Slides were mounted with anti-fade mounting solution (Electron Microscopy Sciences, PA, USA) and observed under an IX81 Olympus microscope with 60X oil immersion objective powered by 1.6x magnification. The images were captured by an ORCA R2 CCD mono camera and processed by the Metamorph Image Analysis Software (Olympus, Japan).

## Results

### DNA-PKcs but not Ku70 is involved in the type I IFN response to CpG-ODNs

**Fig 1:**
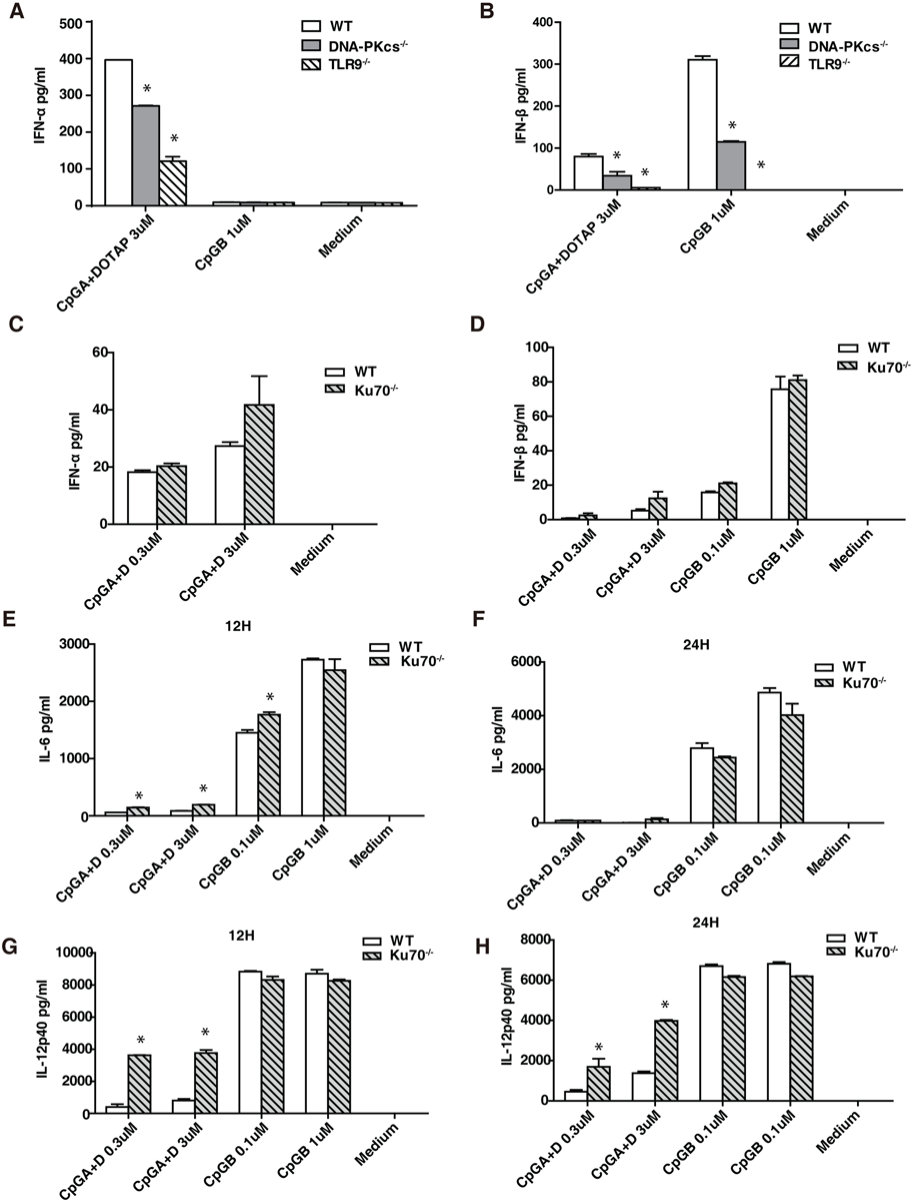
DNA-PKcs but not Ku70 is involved in the type I IFN response to CpG-ODNs. Bone marrow was isolated from WT, DNA-PKcs^-/-^, TLR9^-/-^ and Ku70^-/-^ mice and then cultured with GM-CSF for 7 days. (**A-D**) Harvested BMDCs were seeded in 96-well plates at 2x10^5^/well in triplicate, treated with CpG-ODNs (CpG-A 0.3μM or 3μM with DOTAP; CpG-B 0.1μM or 1μM), or left untreated for 24 hours. The levels of IFNα (**A, C**) and IFNβ (**B, D**) were determined by ELISA. Similar results were obtained in at least three independent experiments. (**E-H**) BMDCs from WT and Ku70^-/-^ mice were seeded at 1x10^5^/well in triplicate, treated with CpG-A 0.3μM or 3μM with DOTAP, CpG-B 0.1μM or 1μM, or left untreated for indicated time durations. The expression of IL-6 (**E, F**) and IL-12 (**G, H**) was determined by ELISA using IL-6 or IL-12 ELISA kits. Similar results were obtained in three independent experiments. Note: bars represent the average of triplicates ± SD. *P< 0.01 KO groups versus WT groups.

We previously reported that DNA-PKcs regulated the IL-6 and IL-12 responses to CpG-ODNs in BMDCs in a time- and dose-dependent fashion[15]. It was also suggested that DNA-PKcs regulated the type I IFN response to CpG-B in a bioassay[13]. Because the bioassay cannot distinguish the anti-viral effect triggered by IFNα or IFNβ, it is unclear whether DNA-PKcs regulates the IFNα or IFNβ response, or both IFNα and IFNβ responses to CpG-B. Thus, we examined secretion of IFNα or IFNβ from WT and DNA-PKcs-deficient BMDCs in response to CpG-A together with DOTAP or CpG-B. As shown, CpG-A induced the secretion of both IFNα and IFNβ, whereas CpG-B only induced the secretion of IFNβ (Fig. 1A and 1B). Loss of DNA-PKcs impaired the IFNα and IFNβ responses to CpG-A, and reduced the IFNβ response to CpG-B (Fig. 1A and 1B). In contrast, loss of Ku70 had no significant effects on the IFNα and IFNβ responses to CpG-A or CpG-B (Fig. 1C to 1D). Loss of Ku70 significantly enhanced the IL-6 and IL-12 responses to CpG-A, but had no significant effects on the IL-6 and IL-12 responses to CpG-B (Fig. 1E to 1H). As a control, we also examined the IFNα and IFNβ responses to CpG-ODNs in TLR9-deficient BMDCs. As expected, loss of TLR9 severely abrogated the IFNβ response to CpG-A and abolished the IFNβ response to CpG-B, but reduced the IFNα response to CpG-A to 1/3 in BMDCs (Fig. 1A and 1B).

Taken together, our data suggest that DNA-PKcs but not Ku70 is important for the type I IFN response to CpG-ODNs.

### Both DNA-PKcs and TLR9 contribute to the IFNα response to CpG-A

**Fig 2:**
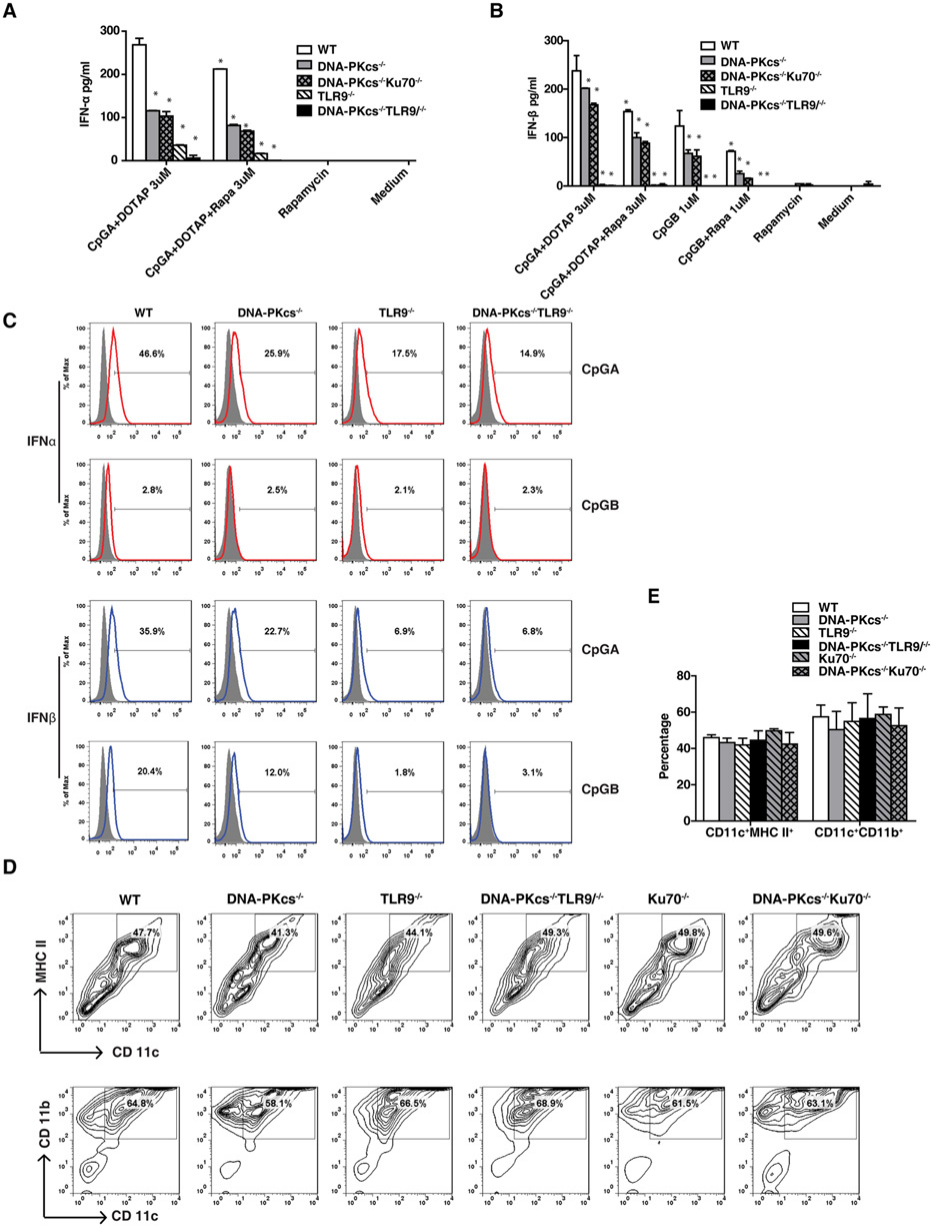
Both DNA-PKcs and TLR9 are important for the IFNα response to CpG-A, and rapamycin diminishes this response. (**A, B**) BMDCs from WT, DNA-PKcs^-/-^, TLR9^-/-^, DNA-PKcs^-/-^/TLR9^-/-^ and DNA-PKcs^-/-^/Ku70^-/-^ mice were cultured for 7 days. Cells were seeded in 96-well plates at 2x10^5^/well in triplicate, and then treated with CpG-A (3μM) plus DOTAP or CpG-B (1μM) in the presence or absence of 100 ng/ml of rapamycin for 24 hours. The levels of IFNα (**A**) and IFNβ (**B**) were determined by ELISA. These experiments were repeated two times, and similar results were obtained. Note: bars represent the average of triplicates ± SD. *P< 0.01 versus WT groups. (**C**) After 7 days culture, BMDCs from WT, DNA-PKcs^-/-^, TLR9^-/-^ and DNA-PKcs^-/-^/TLR9^-/-^ mice were seeded in 3.5 cm petri-dishes at 1x10^7^/dish, and then treated with CpG-A (3μM) plus DOTAP or CpG-B (1μM) for 24 hours. The cells were harvested and stained with anti-CD11b, CD11c and MHC-II Abs followed by internal staining for IFNα or IFNβ. The samples were analyzed by flow cytometry and CD11c^+^CD11b^+^MHC-II^+^ cells were gated and used for the IFN expression comparison. (**D**) BMDCs at day 7 were stained for CD11b, CD11c and MHC-II, and analyzed by flow cytometry. The live cells were gated. (**E**) The percentage of CD11c^+^MHC-II^+^ and CD11c^+^CD11b^+^ BMDCs was analyzed by one-way ANOVA and Tukey’s multiple comparisons test. Note: bars represent the average of three independent experiments ± SD.

Our previous study suggested that DNA-PKcs might be parallel to TLR9 in CpG-DNA signaling[8, 15]. Thus, we determined the type I IFN response to CpG-ODNs in DNA-PKcs/TLR9 double knockout (DKO) BMDCs. Consistent with the results in Fig. 1A, loss of DNA-PKcs or TLR9 reduced the IFNα response to CpG-A (Fig. 2A). However, loss of both DNA-PKcs and TLR9 further reduced this response (Fig. 2A) indicating that DNA-PKcs may be parallel to TLR9 in CpG-A signaling. We also generated DNA-PKcs/Ku70 DKO mice and found that the IFNα and IFNβ responses to CpG-A were not further reduced in DNA-PKcs/Ku70 DKO BMDCs as compared to DNA-PKcs-deficient BMDCs (Fig. 2A, 2B and 2C). Consistent with our previous report[15], loss of DNA-PKcs, Ku70 or TLR9 had no apparent effect on BMDC development or maturation as indicated by cell surface expression of CD11c/MHC-II and CD11b/CD11c (Fig. 2D and 2E). Collectively, our data suggest that both DNA-PKcs and TLR9 are important for the IFNα and IFNβ responses to CpG-ODNs.

### mTORC1 inhibition further diminishes the type I IFN response to CpG-ODNs in DNA-PKcs- and TLR9- deficient BMDCs

It was previously suggested that mTORC1 inhibitor rapamycin impaired the type I IFN response to CpG-ODNs in pDCs[5]. To determine if rapamycin also blocks this response in cDCs (e.g. GM-CSF-driven BMDCs in this study), we treated BMDCs with CpG-ODNs in the presence or absence of rapamycin. Similar to pDCs, rapamycin inhibited the IFNα and IFNβ responses to CpG-A and the IFNβ response to CpG-B in WT BMDCs. Rapamycin further reduced the IFNα response to CpG-A in DNA-PKcs-, DNA-PKcs/Ku70- or TLR9-deficient BMDCs, and almost abolished this response in DNA-PKcs/TLR9 DKO BMDCs (Fig. 2A). Rapamycin additionally diminished the IFNβ response to CpG-A and CpG-B in DNA-PKcs- or DNA-PKcs/Ku70-deficient BMDCs (Fig. 2B). Overall, our data suggest that the residual type I IFN response to CpG-ODN in DNA-PKcs or TLR9-deficient BMDCs is, at least in part, due to the residual activation of mTORC1 by CpG-ODNs.

### The type I IFN is involved in the IL-6 and IL-12 responses to CpG-ODNs in DNA-PKcs-deficient BMDCs

**Fig 3:**
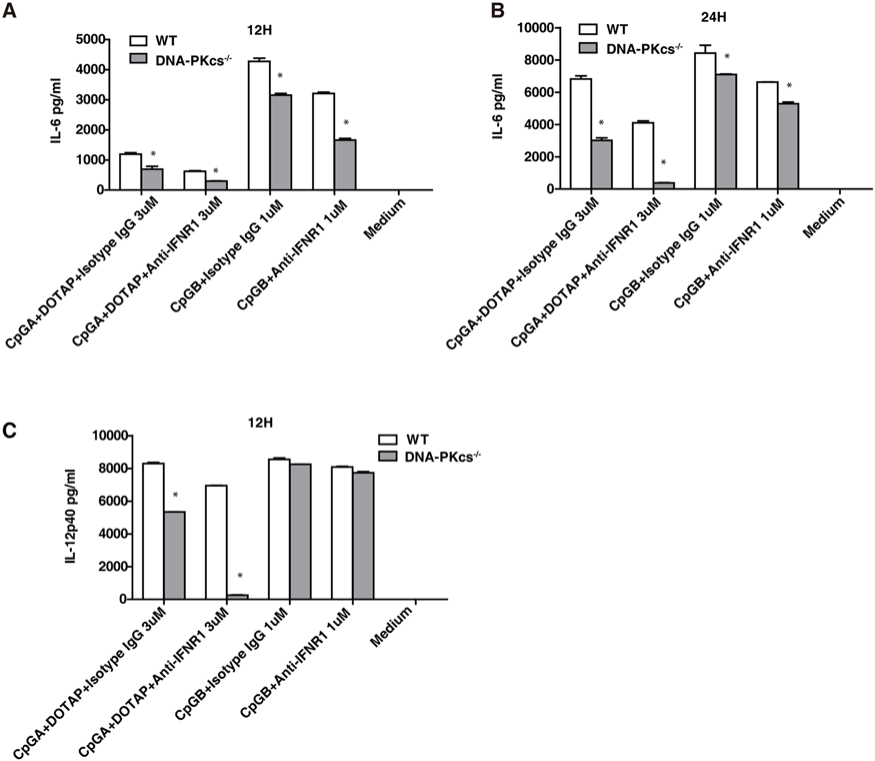
The type I IFNs are involved in the IL-6 and IL-12 responses to CpG-ODNs in DNA-PKcs-deficient DCs. (**A-C**) BMDCs from WT and DNA-PKcs^-/-^ mice were harvested on day 7, and then seeded in 96-well plates at 2x10^5^/well in triplicate. The cells were pre-incubated with 5 μg/ml of anti-IFNAR1 or isotype control Abs for 30 minutes, and then treated with CpG-A (3μM) plus DOTAP, or CpG-B (1μM), or left untreated for 12 or 24 hours. The supernatants were collected and the levels of IL-6 (**A, B**) and IL-12p40(**C**) were detected by ELISA. Similar results were obtained in three independent experiments. Note: bars represent the average of triplicates ± SD. *P< 0.01 versus WT groups.

It was previously reported that the type I IFN contributes to the inflammatory cytokine responses to TLR ligands[19]. Because the type I IFN response (Figs. 1 and 2) and the IL-6 and IL-12 responses to CpG-ODNs were reduced[15], but not abolished in DNA-PKcs-deficient BMDCs, we reasoned that inhibition of type I IFN signaling would further reduce the IL-6 or IL-12 responses to CpG-ODNs in DNA-PKcs-deficient BMDCs. To test this scenario, we treated both WT and DNA-PKcs-deficient BMDCs with CpG-ODNs in the presence or absence of anti-IFNα/β receptor 1 (IFNAR1) neutralizing antibodies. Anti-IFNAR1 antibodies further significantly reduced the IL-6 response to CpG-A and CpG-B (Fig. 3A and 3B) and the IL-12 response to CpG-A in DNA-PKcs-deficient BMDCs (Fig. 3C). In contrast, anti-IFNAR1 antibodies failed to decrease the IL-12 response to CpG-B in either WT or DNA-PKcs-deficient BMDCs (Fig. 3C). Taken together, our data suggest that IFNAR1 signaling contributes to residual pro-inflammatory cytokine response to CpG-ODNs in DNA-PKcs-deficient BMDCs.

### DNA-PKcs and TLR9 are important for the activation of the Akt/mTORC1 pathway by CpG-ODN

**Fig 4:**
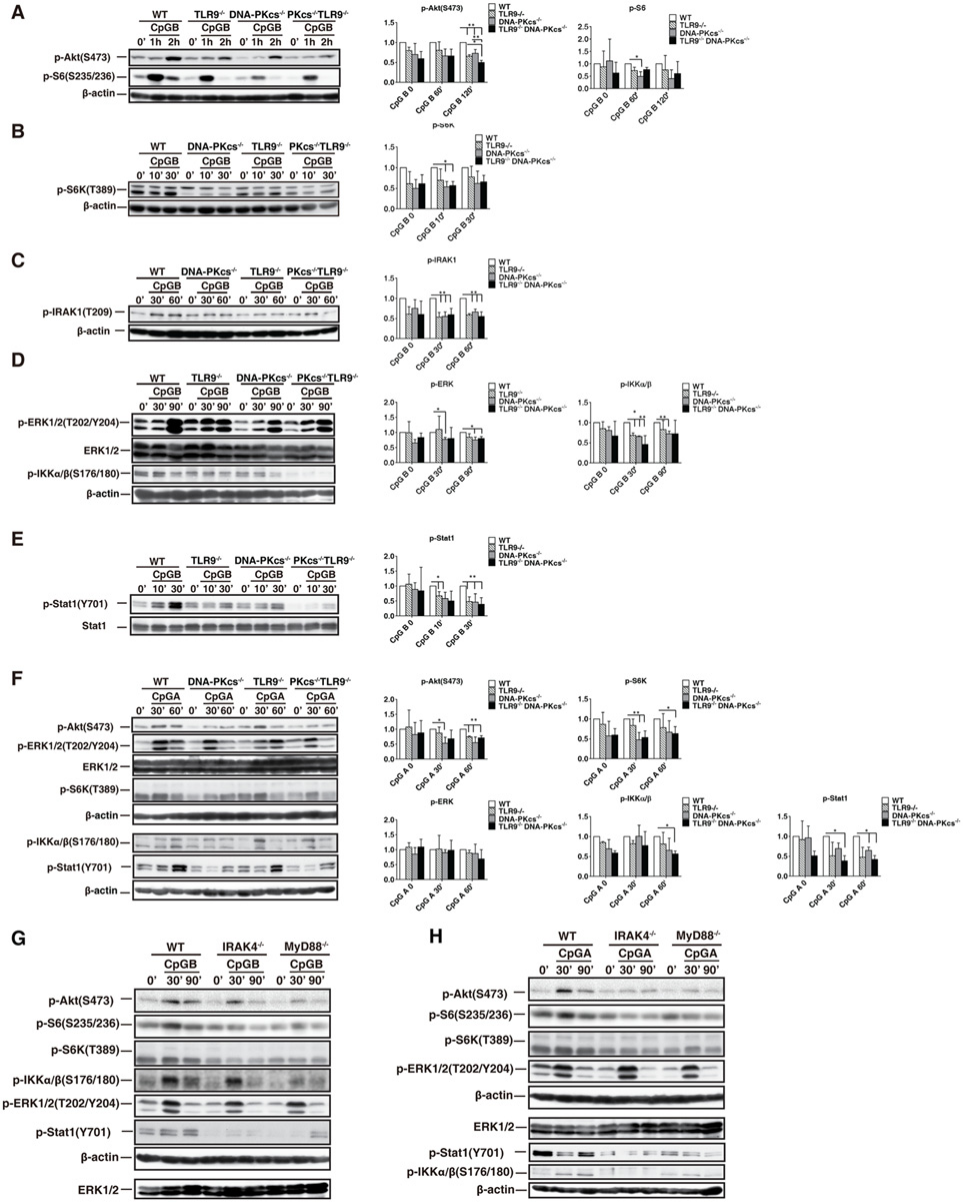
DNA-PKcs and TLR9 are important for phosphorylation of the Akt/mTORC1 pathway, IKKα, IRAK1, ERK and Stat1 in response to CpG-ODN. (**A-F**) WT, DNA-PKcs^-/-^ (PKcs^-/-^), TLR9^-/-^ and PKcs^-/-^/TLR9^-/-^ BMDCs were starved with SFM for 2 hours, and then treated with CpG-ODNs (CpG-B, 1μM or CpG-A, 3μM) for the indicated time durations or left untreated. Cell lysates were prepared and the levels of phosphorylation (p)-Akt(S473), p-S6(S235/236), p-S6K(T389), p-IRAK1(T209), p-ERK1/2(T202/Y204), ERK1/2, p-IKKα/β(S176/180), p-Stat1(Y701) and Stat1 were determined by IB analysis. (**G, H**) BMDCs from WT, IRAK4^-/-^, and MyD88^-/-^ were treated with CpG-B, 1μM or CpG-A, 3μM. The levels of (p)-Akt(S473), p-S6(S235/236), p-S6K(T389), p-ERK1/2(T202/Y204), ERK1/2, p-IKKα/β(S176/180), p-Stat1(Y701) and Stat1 were detected by IB analysis. (**A-D, F-H**) β-actin was used as an equal loading control. Similar results were obtained from three (**A-F**) or two (**G, H**) independent experiments. **p<0.*01*, *p<0.*05*; *Student’s t tes*t. Note: bars represent the average of three independent experiments ± SD.

We previously reported that DNA-PKcs was required, and TLR9 was involved in activation of Akt by CpG-ODN in BMDMs [8]. Also, a study suggested that TLR9 was involved in this activation in BMDMs in response to CpG-B[9]. Because rapamycin inhibits the type I IFN response to CpG-ODNs[5], we determined whether DNA-PKcs and TLR9 are required for activation of the Akt/mTORC1 pathway by CpG-ODNs in BMDCs. As shown, loss of either DNA-PKcs or TLR9 impaired phosphorylation of Akt(S473), S6(S235/236), or S6K(T389) in response to CpG-B (Fig. 4A and 4B); loss of both DNA-PKcs and TLR9 further reduced Akt(S473) phosphorylation (Fig. 4A). Statistically, for Akt(S473) phosphorylation, the significant difference between WT and KO cells occurred at 120 minutes after CpG-ODN stimulation; for S6(S235/236) phosphorylation, the significant difference occurred at 60 minutes; for S6K phosphorylation, the significant difference occurred at 10 minutes.

A similar defect in the above phosphorylation was observed in DNA-PKcs-, TLR9- or DNA-PKcs/TLR9- deficient BMDCs in response to CpG-A (Fig. 4F) except that DNA-PKcs/TLR9 double deficiency did not further significantly impair Akt(S473) phosphorylation. Taken together, our results suggest that both DNA-PKcs and TLR9 are important for the activation of the Akt/mTORC1 pathway in BMDCs by CpG-ODNs.

### DNA-PKcs is important for phosphorylation of IRAK1, IKKα, and Stat1 in response to CpG-ODNs

To further understand how DNA-PKcs is involved in the type I IFN response to CpG-ODNs, first, we determined IKKα and IRAK1 activation, which is required for the type I IFN response to CpG-ODNs[20, 21], in DNA-PKcs-, TLR9- or DNA-PKcs/TLR9- deficient BMDCs. As shown in Fig. 4C, 4D and 4F, loss of either DNA-PKcs or TLR9 reduced IKKα/β(S176/180) and IRAK1(T209) phosphorylation. This reduction became more severe in DNA-PKcs/TLR9 DKO BMDCs in response to CpG-ODNs but was not significant as compared to individual deficient cells. Consistent with a previous report[22], loss of DNA-PKcs reduced ERK1/2(T202/Y204) phosphorylation in response to CpG-ODNs (Fig. 4D). However, clear residual activation of ERK1/2 by CpG-ODNs was observed in DNA-PKcs KO, TLR9 KO, or DNA-PKcs/TLR9 DKO BMDCs (Fig. 4D and 4F), indicating that other protein factors are involved in the ERK1/2 activation by CpG-ODNs.

Finally, we examined phosphorylation of signal transducer activator of transcription 1 at the tyrosine (Y) 701 residue [Stat1(Y701)], which is a key transcription factor in type I IFN signaling[3], in above deficient BMDCs. Loss of either DNA-PKcs or TLR9 reduced Stat1(Y701) phosphorylation (Fig. 4E and 4F), and loss of both DNA-PKcs and TLR9 further diminished Stat1(Y701) phosphorylation in response to CpG-B (Fig. 4E). The most significant difference for the Stat1 phosphorylation between WT, single KO and DKO cells occurred at 30 minutes. Taken together, our data suggest that DNA-PKcs and TLR9 are important for activation of IKKα, IRAK1 and Stat1 by CpG-ODNs.

### MyD88 and IRAK4 are involved in activation of the Akt/mTOR1 pathway by CpG-ODNs

To elucidate the relationship between the TLR9 pathway and the Akt/mTORC1 pathway, we examined phosphorylation of Akt, S6K and S6 in BMDCs generated from mice with deletion of MyD88 or IRAK4, which are key downstream events of TLR9 in CpG-DNA signaling. Our results showed that loss of either IRAK4 or MyD88 reduced but not abolished phosphorylation of Akt(S473), S6K and S6 in BMDCs in response to CpG-ODNs (Fig. 4G and 4H). Also, the levels of phosphorylation of IKKα/β, ERK1/2 or Stat1 were determined. Interestingly, while phosphorylation of IKKα/β and Stat1 was severely impaired in both IRAK4- and MyD88- deficient BMDCs, phosphorylation of ERK1/2 was not markedly reduced (Fig. 4G and 4H), indicating that other molecules but not components in the TLR9 pathway are required for ERK1/2 activation by CpG-ODNs. Taken together, our results suggest that, in addition to their roles in activation of signaling evens in the IFN signaling pathway, IRAK4 and MyD88 are also involved in activation of the Akt/mTOCRs pathway by CpG-ODNs.

### The level of DNA-PKcs is reduced in TLR9-deficient BMDCs

**Fig 5:**
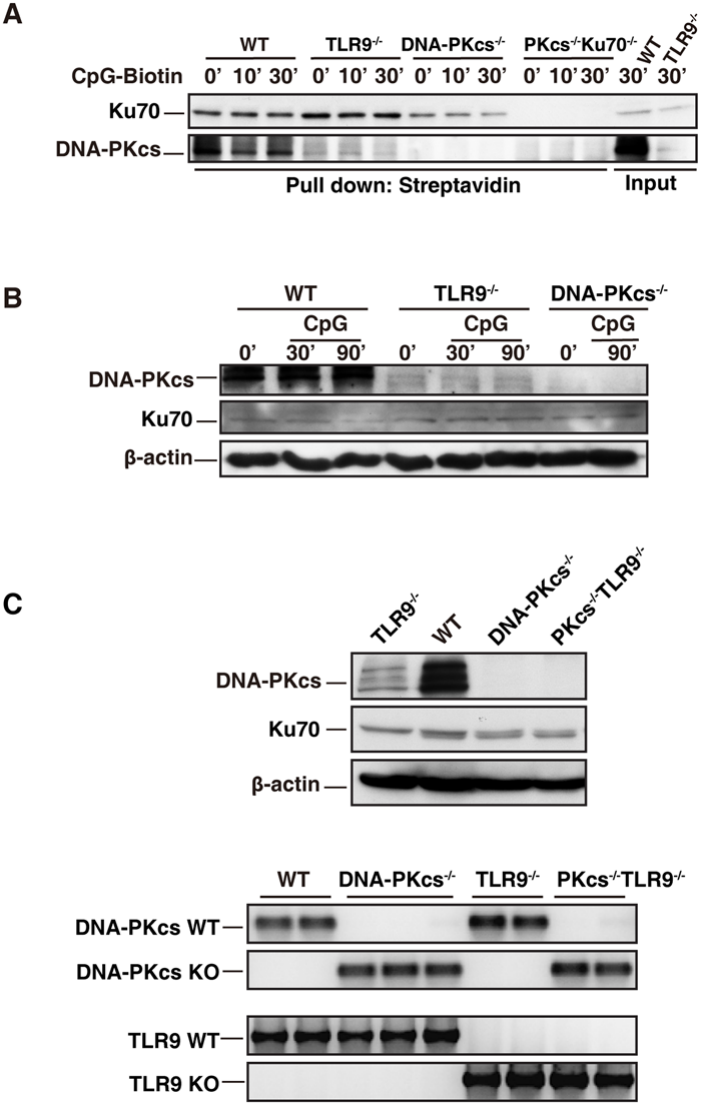
DNA-PKcs level is reduced in TLR9-deficient BMDCs. (**A**) CpG-ODN associates with Ku70 and DNA-PKcs in BMDCs. WT, TLR9^-/-^, DNA-PKcs^-/-^ (PKcs^-/-^), and PKcs^-/-^/Ku70^-/-^ BMDCs were treated with CpG-ODN-biotin (CpG-B-biotin, 1μM) for the indicated time durations. WCLs were prepared with the lysis buffer (1% digitonin). After pre-cleared with protein A/G beads, 400 μg proteins of WCLs were incubated with 15 μg of CpG-B-biotin for 2 hours, and then incubated with 50 μl of streptavidin-agarose beads at 4°C overnight with rotation. The agarose beads were washed four times with the lysis buffer (0.1% digitonin). The samples were separated on 10% SDS-PAGE and transferred onto a PVDF membrane followed by IB with anti-Ku70 or anti-DNA-PKcs Abs. Similar results were obtained from three independent experiments. (**B**) The level of DNA-PKcs is reduced in TLR9-dificient BMDCs. BMDCs from WT, DNA-PKcs^-/-^ (PKcs^-/-^) and TLR9^-/-^ mice were starved in SFM for 2 hours and then treated with CpG-ODN (CpG-B, 1μM) for the indicated time durations or left untreated. Cell lysates were prepared with lysis buffer (1% Triton-X 100) and the expression levels of DNA-PKcs, Ku70 and β-actin were determined by IB. Similar results were obtained from four independent experiments. (**C**) After genotyping, the splenocytes were isolated form WT, TLR9^-/-^, DNA-PKcs^-/-^ (PKcs^-/-^), and PKcs^-/-^/TLR9^-/-^ mice. The red blood cells were lysed and the rest cells were washed twice with PBS. Cell lysates were prepared, and DNA-PKcs, Ku70 and β-actin were detected by IB analysis (left panel). Genotyping results for WT, TLR9^-/-^, DNA-PKcs^-/-^ and DNA-PKcs^-/-^/TLR9^-/-^ mice (right panel).

We previously reported that CpG-ODN bound to DNA-PKcs and TLR9[15]. Here, we confirmed that CpG-ODN associated with DNA-PKcs (Fig. 5A). However, less DNA-PKcs was precipitated by CpG-ODN-biotin from TLR9-deficient BMDCs than WT controls (Fig. 5A). Because Ku70 is a regulatory subunit of DNA-PK, we examined whether Ku70 was pulled down by CpG-ODN-biotin and whether less Ku70 was pulled down from TLR9-deficient BMDCs. As shown, CpG-ODN was able to pull down Ku70 (Fig. 5A). Interestingly, the association of CpG-ODN-biotin with Ku70 was slightly enhanced in TLR9-deficient BMDCs (Fig. 5A) indicating that Ku70 may compete with TLR9 for binding to CpG-ODNs. This may explain why loss of Ku70 enhanced the IL-6 and IL-12 responses to CpG-A, which was delivered to the cytoplasm by DOTAP (lipid).

The above observation led us to hypothesize that TLR9 is important for maintaining the protein level of DNA-PKcs. Indeed, immunoblotting analysis showed that loss of TLR9 clearly decreased DNA-PKcs at the protein level, while Ku70 protein level was not affected by loss of DNA-PKcs, TLR9 or both DNA-PKc and TLR9 (Fig. 5B and 5C).

### CpG-ODN induces the association of DNA-PKcs with TRAF3

**Fig 6:**
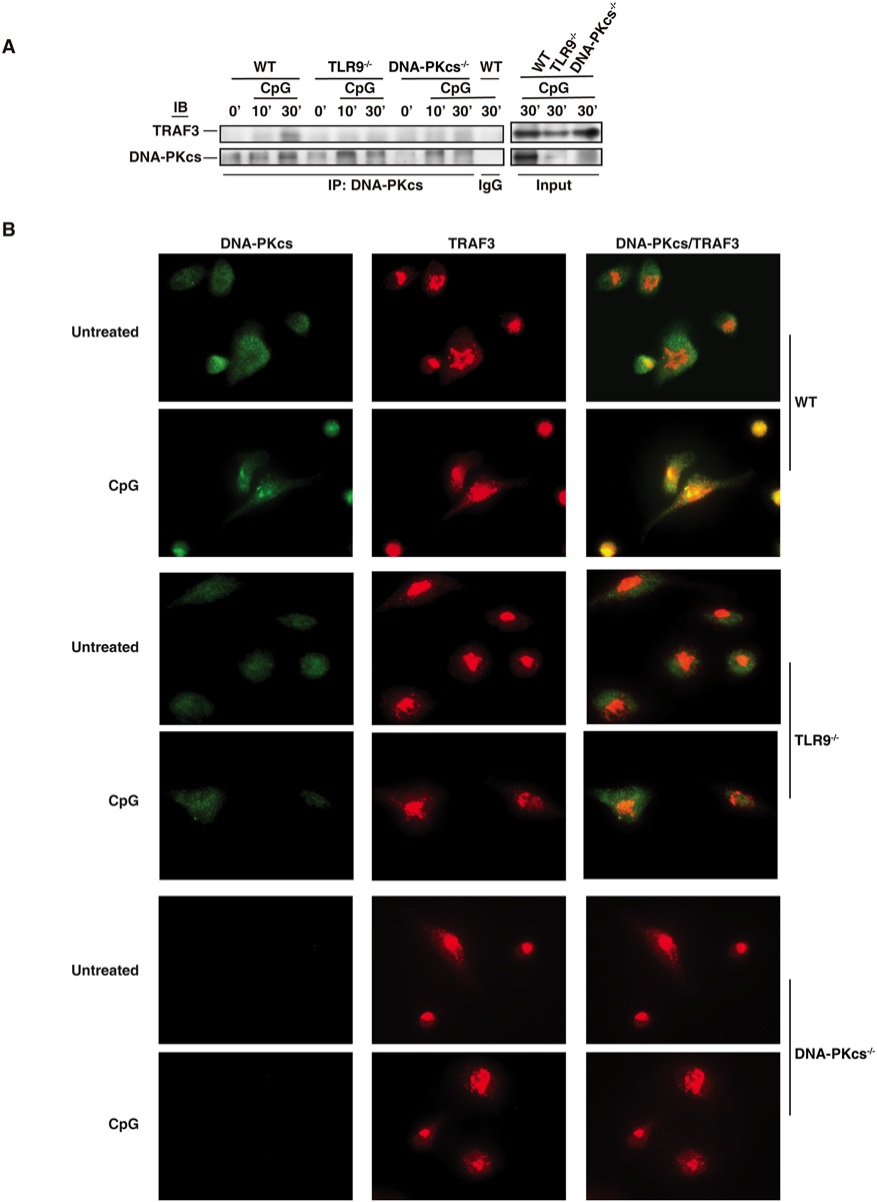
CpG-ODN induces the association of DNA-PKcs with TRAF3. (**A**) WT, TLR9^-/-^, and DNA-PKcs^-/-^ BMDCs were treated with CpG-B (CpG, 1μM) for the indicated time durations. WCLs (400 μg) were prepared with lysis buffer (1% NP-40), pre-cleared with protein A/G beads, and incubated with anti-DNA-PKcs Ab or control IgG and 50 μl of protein A/G beads at 4°C overnight with rotation. Beads were washed four times with the lysis buffer (0.1% NP-40), boiled, loaded onto a 10% SDS-PAGE and transferred onto a PVDF membrane followed by IB assay using anti-TRAF3 or DNA-PKcs Abs. Similar results were obtained from three independent experiments. (**B**) WT, TLR9^-/-^ and DNA-PKcs^-/-^ BMDCs were seeded in an 8-well chamber slide, cultured overnight and then treated with CpG-ODN (CpG-B, 1μM) for 30 minutes or left untreated. The cells were fixed, permeabilized and immunostained with anti-DNA-PKcs Ab (primary)/anti-mouse Alexa 488 (2^nd^ Ab) (green) and anti-TRAF3 Ab (primary)/anti-rabbit Alexa 594 (2^nd^ Ab) (red). Co-localization of DNA-PKcs and TRAF3 were indicated in yellow color in merged images. The cells were observed under an IX81 Olympus microscope with 60X oil objective powered by 1.6x magnification. The images were recorded by an ORCA R2 CCD mono camera and analyzed by the Metamorph advanced for imaging software. Similar results were obtained from two independent experiments.

TRAF3 has been suggested to be required for the type I IFN response to CpG-ODN[3, 4]. Because DNA-PKcs is also involved in this response, we hypothesized that CpG-ODN induces the interaction of DNA-PKcs with TRAF3. To test this hypothesis, we treated WT, DNA-PKcs- or TLR9-deficient BMDCs with CpG-ODN and then immunoprecipitated DNA-PKcs from WT and deficient BMDCs. As shown, CpG-ODN induced the association of DNA-PKcs with TRAF3 (Fig. 6A). Loss of TLR9 largely impaired CpG-ODN-induced association of DNA-PKcs with TRAF3 (Fig. 6A). To confirm the above observation, we performed an immunofluorescence assay. More DNA-PKcs was observed in WT BMDCs compared to TLR9-deficient BMDCs. Much less co-localization of DNA-PKcs with TRAF3 was observed in TLR9-deficient BMDCs in response to CpG-ODN (Fig. 6B). Taken together, our results suggest that CpG-ODN induced the association of DNA-PKcs with TRAF3 in a TLR9- dependent manner.

## Discussion

**Fig 7:**
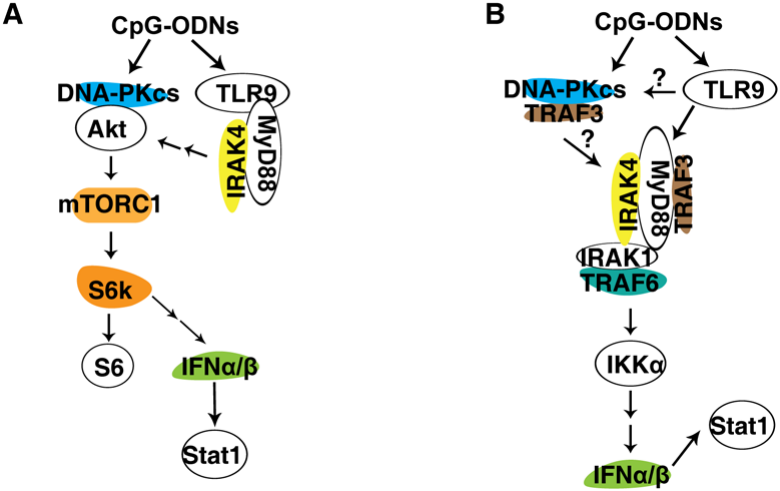
Models of the involvement of DNA-PKcs and TLR9 in the IFNα/β pathway. **(A) DNA-PKcs and TLR9 are involved in activation of Akt and mTORC1 by CpG-ODN.** In response to CpG-ODN, DNA-PKcs associates with Akt, leading to its phosphorylation and activation of downstream event mTORC1, which phosphorylates S6K, which in turn phosphorylates S6. S6K may involve expression of IFNα/β induced by CpG-ODN. Also, CpG-ODN binds and activates TLR9, which in turn recruits MyD88 and IRAK4 leading to activation of Akt and mTORC1. **(B) DNA-PKcs and TLR9 are important for the IFNα/β response to CpG-ODN**. In response to CpG-ODN, DNA-PKcs associates with TRAF3 and activates IRAK1, leading to activation of IKKα and subsequent secretion of IFNα/β, which activate Stat1. Also, in response to CpG-ODN, TLR9 recruits MyD88, which in turn recruits and activates TRAF3 and IRAK4. Activated IRAK4 phosphorylates IRAK1, which then recruits TRAF6 and phosphorylates IKKα, leading to expression of IFNα/β and phosphorylation of Stat1.

TLR9 was thought to be the sole receptor for CpG-ODNs until it was found that a significant amount of CpG-A-induced IFNα was still left in TLR9-deficinet pDCs[23]. Yet, loss of MyD88 and IRAK4, which are key downstream events of TLR9, abolished the IFNα response to CpG-A[23, 24], indicating that other cytoplasmic proteins exist and transduce signal from CpG-A to MyD88 and IRAK4. However, additional molecules involved in the IFNα response to CpG-A remain to be identified. Here, we demonstrate that DNA-PKcs but not Ku70 is also involved in the type I IFN response to CpG-ODNs. Loss of DNA-PKcs reduced the type I IFN response to CpG-A and the IFNβ response to CpG-B. Loss of both DNA-PKcs and TLR9 further diminished the IFNα response to CpG-A, suggesting that both DNA-PKcs and TLR9 are important for the IFNα response to CpG-A (Fig. 7A and 7B).

A previous study suggested that mTORC1 inhibitor rapamycin inhibited the type I IFN response to CpG-A in pDCs and that S6K was required for the interaction of TLR9 with MyD88 and the subsequent IFN response to CpG-ODNs[5]. In this study, we found rapamycin not only inhibited the IFNα response to CpG-A in WT BMDCs, but it also further reduced the IFNα response in either DNA-PKcs- or TLR9-deficient BMDCs (Fig. 2A and 2B), suggesting that mTORC1 activity, at least in part, remains in either deficient cells. We previously reported that loss of DNA-PKcs impaired Akt activation by CpG-ODNs in BMDMs[8]. In BMDCs, loss of either DNA-PKcs or TLR9 reduced Akt(S473) phosphorylation in response to CpG-ODNs suggesting that both DNA-PKcs and TLR9 contribute to the activation of Akt by CpG-ODNs (Figs. 4A and 7A). Similarly, loss of DNA-PKcs or TLR9 reduced phosphorylation of S6K and S6 in response to CpG-ODNs (Fig. 4A and 4B). Our results explain why rapamycin further inhibited the IFNα response to CpG-A in either DNA-PKcs- or TLR9-deficient BMDCs. To further understand how TLR9 is involved in activation of Akt and mTORC1 by CpG-ODN, we examined this activation in IRAK4- or MyD88-deficient BMDCs. Loss of either IRAK4 or MyD88 reduced but not abolished phosphorylation of Akt, S6K and S6, thus suggesting that the activation of the Akt/mTORC1 pathway by TLR9 is mediated by its downstream events MyD88 and IRAK4 (Figs. 4G, 4H, and 7A).

IRAK1 and IKKα are required for CpG-ODN-induced type I IFN expression[3]. Our data showed that IRAK1 and IKKα activation was more defective in DNA-PKcs/TLR9 DKO BMDCs than that in individual KO BMDCs (Fig. 4C and 4D). Our results further explain why the IFNα response to CpG-ODN was more severely impaired in DNA-PKcs/TLR9 DKO BMDCs than in either DNA-PKcs- or TLR9-deficient BMDCs. Additionally, CpG-ODN-induced Stat1(Y701) phosphorylation was more severely reduced in DNA-PKcs/TLR9 DKO BMDCs than in individual deficient BMDCs (Fig. 4E).

Rapamycin and the blockade of the type I IFN signaling inhibited the pro-inflammatory cytokine response to CpG-ODN[5]. We found that rapamycin and anti-IFNAR1 antibodies not only inhibited the IL-6 and IL-12 responses to CpG-ODN in WT BMDCs, but they also further decreased this response in DNA-PKcs-deficient BMDCs (Fig. 2A and 2B, and data not shown). Because DNA-PKcs is also involved in activation of the Akt/mTORC1 pathway and the type I IFN response to CpG-ODN, our data suggest that the involvement of DNA-PKcs in the IL-6 and IL-12 responses to CpG-ODN is partially due to its contribution to the type I IFN response and the activation of the Akt/mTORC1 pathway.

We previously reported that CpG-ODN-biotin was able to precipitate both DNA-PKcs and TLR9[15]. Here, we showed that CpG-ODN-biotin was able to pull down DNA-PKcs and Ku70 (Fig. 5A). However, loss of TLR9 reduced the precipitated amount of DNA-PKcs by CpG-ODN, but this deficiency seemed to enhance Ku70 pull-down (Fig. 5A). Three possible situations may lead to this phenomenon. First, because DNA-PKcs can form a complex with Ku70[10], it is very possible that Ku70 competes with DNA-PKcs for binding to CpG-ODN in the absence of TLR9. The second possibility is that TLR9 assists in DNA-PKcs’s binding to CpG-ODN or its association with Ku70. The third possibility is that DNA-PKcs is less stable or less expressed in TLR9-deficient BMDCs. Because in WT BMDCs CpG-ODN efficiently pulled down both DNA-PKcs and Ku70, it is less likely that the absence of TLR9 will strikingly decrease DNA-PKcs-CpG-ODN binding. Also, DNA-PKcs is a cytoplasmic protein in mouse cells[8, 15] and its distribution is not limited to the endosome or lysosome compartments. It is less likely that TLR9 will stimulate DNA-PKcs’s binding to CpG-ODN or its interaction with Ku70. Thus, we favor the third possibility that TLR9 may control DNA-PKcs expression or stability. Indeed, we found that loss of TLR9 clearly reduced DNA-PKcs protein level (Fig. 5B and 5C). The reduction of DNA-PKcs in TLR9-deficient BMDCs always occurred, but the degree of the reduction varied reflecting the nature of primary BMDC culture with growth factors or a plausible alteration of activities of protease inhibitors provided to cells when they were lysed each time. Nevertheless, the molecular mechanism of how TLR9 deficiency leads to a low level of DNA-PKcs in BMDCs remains elusive.

TRAF3 is critical for the type I IFN response to TLR ligands including CpG-ODN[3, 4]. TRAF3 associates with MyD88 and TRIF, and loss of TRAF3 severely impairs production of type I IFN in response to CpG-ODN, poly(I:C), R848 (TLR7/8) and lipopolysaccharide [4]. Our data showed that the association of DNA-PKcs with TRAF3 was clearly induced by CpG-ODN in WT but not in TLR9-deficient BMDCs (Fig. 6A and 6B). The later could be due to reduced expression of DNA-PKcs in TLR-deficient cells. However, it is also possible that TLR9 is indeed required for the interaction of DNA-PKcs with TRAF3 in response to CpG-ODNs. Nonetheless, this finding further supports our conclusion that DNA-PKcs is involved in the type I IFN response to CpG-ODNs.

In summary, DNA-PKcs and TLR9 are important for the type I IFN response to CpG-ODNs. Both DNA-PKcs and TLR9 contribute to the IFNα response to CpG-A, whereas TLR9 is essential for the IFNβ response to CpG-ODNs. These IFN responses involve both the MyD88/IRAK4/IRAK1/IKKα and the Akt/mTORC1/S6K pathways (Fig. 7A and 7B). Therefore, DNA-PKcs regulates the type IFN response to CpG-ODNs in TLR9-dependent or independent manners.
